# Warfare: NRDC Knocks Nukes

**DOI:** 10.1289/ehp.112-a984

**Published:** 2004-12

**Authors:** Ron Chepesiuk

More than a decade after the end of the Cold War, the United States is spending 12 times as much on nuclear weapons and production as it does on programs to find and dispose of nuclear weapons, according to a recent report by the Natural Resources Defense Council (NRDC), a Washington, D.C.–based environmental organization. The report warns that the continued push for a new generation of nuclear weapons could lead to a second arms race. And with another arms race, analysts fear, could come renewed nuclear weapons testing and potential serious fallout for the environment.

“There is no good reason why the U.S. should be spending on average more than it did during the Cold War,” says Chris Paine, an analyst with the NRDC’s Nuclear Program and the report’s author. “The U.S. government needs to rethink the role that nuclear weapons should play in the post–Cold War era.”

Titled *Weaponeers of Waste*, the report analyzes six nuclear weapons projects, primarily located at the Los Alamos, Sandia, and Lawrence Liver-more laboratories. According to the report, the government spent $6.5 billion in fiscal year 2004, compared to the average $4.2 billion dollars (in 2004 dollars) it spent yearly during the Cold War. In fiscal year 2005, the U.S. Department of Energy (DOE), the federal agency overseeing the nuclear weapons complex, is asking Congress for $6.8 billion to support its nuclear weapons projects.

The report describes several of the projects it reviewed as “boondoggles.” They include the huge National Ignition Facility at Lawrence Livermore National Laboratory, a high-energy fusion laser that in 1997 the DOE said would be ready in 2005 at a cost of $1.2 billion, but now, after factoring in additional expenses and recalculating construction management costs, will cost as much as $5–8 billion. The government expects to complete the facility sometime between 2010 and 2014.

“The government seems determined to put a lot of money into very expensive nuclear weapons projects at the expense of nonproliferation programs, which are hurting for funding,” says Victoria Samson, a research analyst with the Washington, D.C.–based Center for Defense Information, a think tank that monitors the U.S. defense industry. “At a time that we are in a war on terrorism, it doesn’t seem like a good idea to spend money on [nuclear weapons] programs.”

Others believe the report is the real boondoggle. “The NDRC budget assessment is wrong, and they are misleading people,” says Bryan Wilkes, director of public affairs for the National Nuclear Security Administration in Washington, D.C. “Our weapons program budget also includes money for such things as security, administrative costs, infrastructure repair, secure transportation [to move weapons secretly around the country], and emergency response teams—not just nuclear weapons.”

Wilkes says charges that the government is spending too much money on nuclear weapons are irresponsible. He points to the science-based Stockpile Stewardship Program, which conducts tests to ensure the current stockpile remains safe, secure, and reliable without underground testing.

Wilkes further says the government has increased spending on nonproliferation programs by more than 60%. “If that figure doesn’t reflect our priorities,” he says, “I don’t know what does.” And he adamantly maintains there are no plans to develop, produce, or test nuclear weapons.

Yet according to Martin Butcher, director of security programs for the Washington, D.C.–based Physicians for Social Responsibility, there is deep skepticism in some quarters about such assurances, and concern that the Bush administration’s research program on nuclear weapons and refusal to ratify the Comprehensive Nuclear Test Ban Treaty may lead to the development of new or modified weapons that require proof testing.

“The history of nuclear weapons in this country has been an environmental catastrophe,” Butcher says. “We don’t want to repeat the mistakes of the past.” He adds that the Bush administration has given no signs of planning to resume atmospheric testing, but underground testing in the Nevada test site would still lead to the venting of irradiated gases. In the 1989 report *The Contamination of Underground Nuclear Explosions*, the congressional Office of Technology reported that since 1970, a total of 126 underground tests have resulted in 54,000 curies of radiation being vented into the atmosphere. Just one accident during nuclear testing could release 150 million curies into the atmosphere—about equivalent to what resulted when the atomic bomb was dropped on Hiroshima during World War II.

In the report, the NDRC urges Congress to implement several recommendations. They include consolidating the nuclear weapons complex to reduce costs, eliminate redundancies, and lessen its environmental footprint. The group also urges putting more focus on international efforts to reduce stockpiles and funding for the preparation of nuclear testing.

“Congress needs to take a closer look at the role and mission of our nuclear weapons programs,” says Daryl Kimball, executive director of the Arms Control Association, an independent organization that supports effective arms control and disarmament policies. “It’s a bad idea to assign new [projects and programs] to our country’s nuclear weapons program.”

## Figures and Tables

**Figure:**
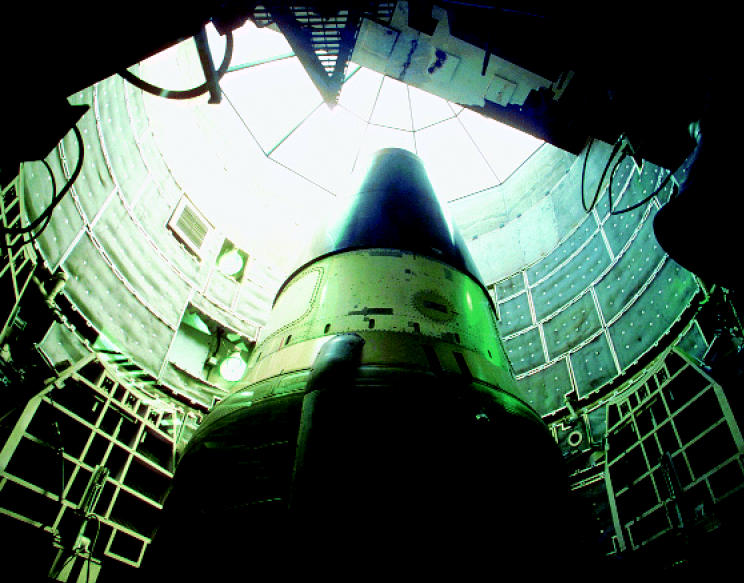
**Taking aim at nukes.** A new report by the Natural Resources Defense Council challenges the government’s spending on and handling of nuclear weapons.

